# Increased Risks of Mental Disorders: Youth with Inactive Physical Activity

**DOI:** 10.3390/healthcare10020237

**Published:** 2022-01-26

**Authors:** Ángel Denche-Zamorano, Juan Manuel Franco-García, Jorge Carlos-Vivas, María Mendoza-Muñoz, Damián Pereira-Payo, Raquel Pastor-Cisneros, Eugenio Merellano-Navarro, José Carmelo Adsuar

**Affiliations:** 1 Promoting a Healthy Society (PHeSO) Research Group, Faculty of Sport Sciences, University of Extremadura, 10003 Cáceres, Spain; angeldenche@gmail.com (Á.D.-Z.); jorgecv@unex.es (J.C.-V.); mamendozam@unex.es (M.M.-M.); raquelpc@unex.es (R.P.-C.); jadssal@unex.es (J.C.A.); 2 Health, Economy, Motricity and Education (HEME) Research Group, Faculty of Sport Sciences, University of Extremadura, 10003 Cáceres, Spain; dpereirab@alumnos.unex.es; 3 Grupo de Investigacion EFISAL, Universidad Autónoma de Chile, Talca 3460000, Chile; emerellano@gmail.com

**Keywords:** anxiety, depression, mental health, physical activity, sedentary lifestyle

## Abstract

Before COVID-19, one of the most dangerous pandemics of the 21st century was physical inactivity (PI). Sedentary habits had increased in the last decades, reducing physical condition and increasing non-communicable diseases and mental disorders in the population. This study aimed to analyse the relationships between physical activity level (PAL) and the prevalence of anxiety, depression, and other mental disorders in Spanish young aged 15–35 years and to calculate the odd ratio (OR) of developing from these mental disorders in inactive young people, based on PAL. Methods: A cross-sectional study based on data from the Spanish National Health Survey 2017 with 4195 participants was conducted. A descriptive analysis was performed. Possible differences between groups were analysed using the non-parametric statistical tests. OR and relative risks for mental disorders in inactive versus others PAL were calculated. Results: Dependence relationships were found between PAL and the prevalence of anxiety, depression, and other mental disorders (*p* < 0.001). In addition, the risk of developing: anxiety (OR: 6.14. 95% CI: 3.28–11.50), depression (OR: 5.35. 95% CI: 2.40–11.96), and other mental disorders (OR: 8.52. 95% CI: 2.90–25.06) was higher in inactive young people. Conclusions: PI is associated to high risk of mental disorders in Spanish young people.

## 1. Introduction

Before COVID-19, physical inactivity was considered one of the most dangerous pandemics of the 21st century [1,2]. In the pre-pandemic period, sedentary behaviours were increasing in prevalence, becoming part of people’s lifestyle in modern societies. Insufficient physical activity had a global prevalence of 27.5%, and the biggest numbers were registered in Latin America, South Asia, and Western countries [3].

The emergence of SARS-CoV-2 and all the measures taken by governments and agencies to counteract its spread have meant that sedentary habits and physical inactivity have increased even more in the last two years, reaching between 70–80% of physical inactivity prevalence in some countries, such as Iran or Brazil [4,5].

The emergence of new technologies, such as the use of mobile devices, greater accessibility to internet connections, new equipment, increased screen time, less active journeys, more sedentary jobs, city lifestyles, lack of time, economic and employment insecurity, lack of access to infrastructure, and a long etcetera, are some of the causes of the growth in physical inactivity and some of the barriers identified for the lack of physical activity in the population [6,7].

The negative effects of new technologies on people’s health have been demonstrated, such as reduced physical fitness, increased prevalence of overweight and obesity, and increased prevalence of non-transmissible diseases, including an increase in mental health conditions [8,9,10].

### Theoretical Framework: Physical Inactivity and Mental Disorders

Worldwide, 14% of young people and adolescents presented some type of mental health problem in 2020, with anxiety and depression being the most prevalent pathologies [11]. In Spain, it is estimated that in the adult population, the prevalence of any type of mental disorder across the life cycle is about 10–20% [12,13].

Anxiety is the most common mental health problem in youth, affecting 12–20% of young population worldwide. It is associated with sleep disturbance and loss of quality of life [14]. In the Spanish population, 6.7% suffer from anxiety, with these numbers being higher in women than in men (9.2% vs. 4%) [15].

Depression is the second most prevalent mental problem in the world, with an estimated prevalence of 5% in the world population [16,17]. In the Spanish child and youth population, depression had a prevalence of around 4% although with ranges from 1 to 7% [18]. Other mental diseases that have affected the population include bipolar affective disorders, schizophrenia, psychosis, and others although they have lower incidences [19].

Relationships have been found between physical inactivity or sedentary behaviours and unfavourable health conditions [20], reduced quality of life [21], as well as increases in all types of non-communicable diseases, such as heart disease, metabolic syndrome [22,23,24], depression, anxiety, and other mental disorders in young people and adolescents [25,26,27].

Physical inactivity is inversely associated with mental health in both men and women [28] and directly related to psychological distress, depressive symptoms, and lower mental well-being, life satisfaction, and happiness in youth [29].

Conversely, maintaining high levels of physical activity (PA) has been associated with health benefits [30], reducing the prevalence, risks, or deaths from non-communicable diseases [31] and improving health-related quality of life [32], and it has even been identified as a potential tool to reduce symptoms of anxiety and depression in young people and adolescents [33].

Therefore, this study aimed (1) to analyse the relationships between the level of physical activity and the prevalence of chronic anxiety, depression, and other mental disorders in Spanish young people aged 15–35 years and (2) to calculate the risk probability of developing these mental disorders in inactive young people based on their level of physical activity.

Thus, the initial hypotheses were as follows: (1) the prevalence of mental disorders in Spanish youth is related to the level of physical activity, and (2) physical inactivity is associated with an increased risk of developing mental disorders in Spanish youth.

## 2. Materials and Methods

### 2.1. Study Design, Participants, and Ethics

A descriptive cross-sectional study was carried out on the young population included and surveyed in the 2017 Spanish National Health Survey (ENSE 2017) [34], a survey conducted by the Ministry of Health, Consumer Affairs, and Social Welfare (MSCBS), alongside the Spanish National Institute of Statistics (INE), to determine the health status of the Spanish population, which is repeated every five years.

The sample was selected by a stratified three-phase random system, considering people aged 15 years and over who resided in Spain.

A total of 23,860 households were randomly selected by personal interviews with a randomly selected member of the household.

The interviews were carried out by trained and accredited interviewers. The whole national geographic scope was covered, forming a total sample of 29,195 people, of which 23,089 were adults aged 15 and over and were interviewed with the ENSE 2017, an adult questionnaire [35].

In this research, young people of Spanish nationality aged between 15 and 35 years old were included [36,37], obtaining results in all the variables used in this research. The final sample consisted of 4195 people (2013 men and 2182 women).

All data were extracted from the anonymous public files published by the MSCBS and the INE. Prior to the interview, the MCSBS sent a letter requesting the collaboration of the selected persons, informing them of their inclusion in the ENSE 2017 and of the confidential nature of the collection and dissemination of the data.

Regulation (EU) 2016/679 of the European Parliament and Council of 27 April 2016 on the processing of personal data does not consider the type of public files used in this research to be confidential.

The use of these data did not require approval from an accredited ethics committee, as they are not covered by data protection principles, as they are non-identifiable, anonymous data.

### 2.2. Variables

Besides sex and age, which were used to group the sample into males and females and 15–24 years and 25–35 years, two items were used to create the mental health variables according to having or having not experienced: depression, chronic anxiety, and/or other mental disorders.

Items G.25.20a (Do you experience or have you ever experienced depression?) and G.25.20.c (*Has a doctor told you that you have it?*) were used to construct the variable “Depression”, with values: “Yes” if they had responded affirmatively to items G.25.20A and G.25.20C and “No” if they responded negatively to G.25.20A or, with this affirmative, negatively to G.25.20C. Similarly, items G.25.21A (Do you experience or have you ever experienced chronic anxiety?) and G.25.21C (Has a doctor told you that you have it?) and variables G.25.22A (Do you experience or have you ever experienced chronic anxiety?) and G25.22C (Has a doctor told you that you have it?) were used to form the variables “Chronic anxiety” and “Other mental disorders”, respectively.

In addition, a variable was created to group young people according to the Physical Activity (PA) performed, namely “Physical activity level” (PAL). To create this, a PA index (IAF, by its Spanish acronym) was created using an adaptation of the Physical Activity Index (PAI) [38] from the following items: p.113 (How many days did you do vigorous PA?), p.114 (How much time did you dedicate in total to vigorous PA?), p.115 (How many days did you do moderate PA?), and p.116 (How much time did you dedicate in total to moderate PA?). Completing the PAL were the answers to item p.117 (Now, think about the time you dedicated to walking in the last 7 days). The formula for the PAL was IAF = Intense activity score + Moderate activity score, where:

Intense activity score: Intense intensity factor (item p.113 was assigned an intensity factor of 10 for intense activity) * Intense activity frequency factor (responses to item p.113 were assigned the following factors: 0 if the response was “No day per week”; 1, “One day per week”; 2, “Two or three days per week”; or 3, “More than three days per week”) * Factor of time of intense activities (the responses of item p.114 were assigned the following factors: 1, “Less than 30 min”; 1.5, “30 or more minutes”) [38].

Moderate activity score: Moderate-intensity factor (item p.115 was assigned an intensity factor of 5 as moderate activity) * Moderate activity frequency factor (responses to item p.115 were assigned the following factors: 0 if the response was “No day per week”; 1, “One day per week”; 2, “Two or three days per week”; or 3, “More than three days per week”) * Moderate activity time factor (responses to item p.116 were assigned the following factors: 1, “Less than 30 min”; 1.5, “30 or more minutes”) [38].

The IAF scores could range from 0 to 67.5 points. With the scores of the participants in it, the PAL variable was configured, grouping them into the following: Inactives (IAF = 0; reported not walking, at least one day a week more than 10 min at a time on item p.117), Walkers (IAF = 0; reported walking, at least one day a week more than 10 min at a time on item p.117), Actives (IAF between 1 and 30), and Very actives (IAF higher than 30).

### 2.3. Statistical Analyses

All analyses were conducted using IBM SPSS Statistics v.25 software (IBM, Armonk, NY, USA). Data distribution for each variable were analysed using the Kolmogorov–Smirnov test, but the results did not provide sufficient evidence to be able to assume the normality of the data. Thus, median and interquartile range of the age of the population were obtained both for the general population and stratified by sex.

Possible differences between groups were analysed using the non-parametric Mann–Whitney U test. Subsequently, we calculated the relative and absolute frequencies of the variables: age group, depression, chronic anxiety, other mental disorders, and physical activity, both in the general population and by sex, studying relationships of dependence between these and sex by using the chi-square statistic and evaluating possible differences between proportions by using a pairwise z-test.

The same tests were performed to analyse dependency relationships and possible differences in proportions between the level of physical activity and the prevalence of depression, chronic anxiety, and other mental disorders. Finally, odds ratios (OR) and relative risks (RR) were calculated, together with their confidence intervals, between the level of inactive PA and the rest of PA levels in relation to the prevalence of depression, chronic anxiety, and other mental disorders. Significance level was set at *p* ≤ 0.05.

## 3. Results

The sample analysed in this study, which included young people between 15 and 35 years of age from the ENSE 2017, had a median age of 27 years, had equal numbers of men and women, and had no significant differences between sexes (*p =* 0.114). However, we did find dependency relationships between sex and age group (*p* < 0.005), with significant differences in the distribution of sexes in the different age groups, with a higher proportion of males in the 15–24 age range and females in the 25–35 age range (*p* < 0.05) (Table 1).

The prevalence of depression diagnosed in Spanish youth was 3.1%. It was higher in women than in men (4.0% vs. 2.2%. *p* < 0.05). Dependence relationships were found between the prevalence of depression and sex (*p* < 0.005). With higher prevalence, similar findings were shown for chronic anxiety. The prevalence in the sample of Spanish youth analysed was 5.6%, with higher percentages in women than in men (6.1% vs. 3.1%. *p* < 0.05) and with dependency relationships found between sex and the prevalence of chronic anxiety (*p* < 0.001).

Conversely, the prevalence of other mental disorders was higher in men than in women (2.2% vs. 1.2%. *p* < 0.05), and a dependency relationship between sex and the prevalence of other mental disorders was found (*p* < 0.05) (Table 1).

In all, 12.4% of Spanish youth were inactive, with a higher proportion of inactive PA levels in women than in men (13.9% vs. 10.1%. *p* < 0.05).

The PA level with the highest prevalence was the Walker group, with 36% of the population, although it was not the same in both sexes. The differences in proportions between women and men were close to 19 percentage points, and it was lower in men (45.0% vs. 26.3%. *p* < 0.05). In contrast, males had higher prevalence of PA, reaching a prevalence 16 percentage points higher than females at the Very active level (28.7% vs. 12.7%. *p* < 0.05). In addition, dependence relationships were found between sex and PA level (*p* < 0.001) (Table 1).

The prevalence of depression was found to be related to the level of PA of Spanish youth, with dependence relationships found between both variables (*p* < 0.001). Inactive youth had the highest prevalence of all PA groups although no significant differences were found between Inactives and Walkers (4.8% vs. 4.1%); significant differences were found between Inactives, Actives, and Very Actives as well as between these groups and each other (4.8% vs. 2.8% vs. 0.9%. *p* < 0.05). PA was also found to be related to the prevalence of chronic anxiety, showing dependence relationships (*p* < 0.001).

Inactives also presented the highest prevalence of chronic anxiety among all levels of PA, with statistically significant differences with the proportions of the rest of the groups (8.7% vs. 5.5% vs. 4.2%. *p* < 0.05 between Inactives, Walkers, and Actives, respectively), reaching the highest difference in proportions with the Very actives (8.7% vs. 1.5%. *p* < 0.05), which presented significant differences with all groups (*p* < 0.05).

Finally, the prevalence of other mental disorders showed a dependence relationship with the level of PA (*p* < 0.001). The prevalence of other mental disorders was found to be higher in the Inactives than in the other groups, with significant differences with Walkers and Actives and between these and Very actives PA level group but not between them (3.9% vs. 1.7–1.4% vs. 0.5%. *p* < 0.05) (Table 2).

In the young Spanish population, the Inactives group presented higher ORs and RRs compared to the rest of the PA groups in the three mental health conditions analysed: depression, chronic anxiety, and other mental disorders. Although in depression, they were not significant with the Walkers group, the general population, or in the two age groups.

The highest ORs and RRs were found between the Inactives and Very Actives PA levels: depression (OR: 5.35. IC95%: 2.39–11.96. RR: 2.06. IC95%: 1.67–2.52. *p* < 0.001), being even higher in the 25–35 age group; anxiety (OR: 6.14. IC95%: 3.28–11.50. RR: 2.15. IC95%: 1.84–2.52. *p* < 0.001), also being even higher in the 25–35 years age group; and other mental disorders (OR: 8.52. IC95%: 2.90–25.06. RR: 2.25. IC95%: 1.86–2.73. *p* < 0.001). We also found statistically significant higher ORs and RRs between Inactives vs. Walkers in chronic anxiety (OR: 1.63. IC95%: 1.12–2.38. RR: 1.41. IC95%: 1.10–1.81. *p* < 0.05) and other mental disorders (OR: 2.38. IC95%: 1.31–4.33. RR: 1.77. IC95%: 1.26–2.47. *p* < 0.005) (Table 3).

## 4. Discussion

### 4.1. Youth Mental Health Discussion: Differences by Gender

The first results of this research on the mental health of Spanish youth in the pre-pandemic period were the dependence relationship between the prevalence of depression (*p* < 0.005), chronic anxiety (*p* < 0.001), and other mental illnesses (*p* < 0.05) and gender.

The prevalence of depression diagnosis in young people in Spain was 3.1%, which is lower than the prevalence found worldwide, which is estimated to be around 5% in the overall population [17].

Gender seems to have an influence on the mental health of young people. Being female was found to be associated with higher prevalence of depression (4.0% vs. 2.2%), with a ratio of 1.8:1, similar to those found in the scientific literature, where such prevalence ratios of depression in men and women are usually found to be between 1.5:1 and 2:1, with this differentiation occurring from adolescence and consolidating or increasing in adulthood [39].

These findings were also found to be correlated with the prevalence of diagnosed chronic anxiety. In Spanish youth, it was found to be 4.7%, slightly lower than that found in the worldwide population in other studies, at 6.5%. One example is the study by Polanczyk et al. [40], with data from 27 countries [40,41], where it was higher in women than in men (6.1% vs. 3.1%), a difference in prevalence close to double (*p* < 0.05), as it has already been found in other studies reporting that the prevalence of anxiety in women is double that of men [42]. Chronic anxiety, too, has been found to be higher in young women in other research, with some of the main causes being body dissatisfaction and weight control behaviours of young women due to gender bias as well as hormonal differentiations; this phenomenon is not so widespread in men [43,44]. If women seem to be more likely to suffer from depression and anxiety, men seem to be more likely to suffer from other mental illnesses, such as schizophrenia, autism, or attention deficit hyperactivity disorder [45,46]. This was found in the prevalence of other mental disorders in Spanish youth, and results showed that it was higher in males than in females (2.0% vs. 1.2%. *p* < 0.05) in a variable that grouped any mental illness other than anxiety and depression.

### 4.2. Physical Activity Level Discussion: Differences by Gender

The level of PA performed by Spanish youth was also found to be related to sex (*p* < 0.001). A total of 48.4% reported not doing moderate and/or intense PA at least one day a week although differences were found between men and women (37% vs. 58.9%). The proportion of inactive women was 3.2 percentage points higher than men (13.9% vs. 10.7%. *p* < 0.05).

However, the biggest difference was found in the sex distribution at the Walking level, where the proportion of young women walking was much higher than men’s (45% vs. 26.3%. *p* < 0.05); similar results have been found in very different parts of the world, such as Bogotá, London, and Buenos Aires, among others [47]. In contrast, the proportion of men with high levels of PA was higher than women, with 63% of men performing moderate and/or intense PA compared to 41.1% of women. These PA differences may have some bearing on the increased prevalence of depression and chronic anxiety in women, or PA may have helped to reduce this prevalence.

### 4.3. Relationship between Level of Physical Activity and Mental Problems

**Figure 1:**
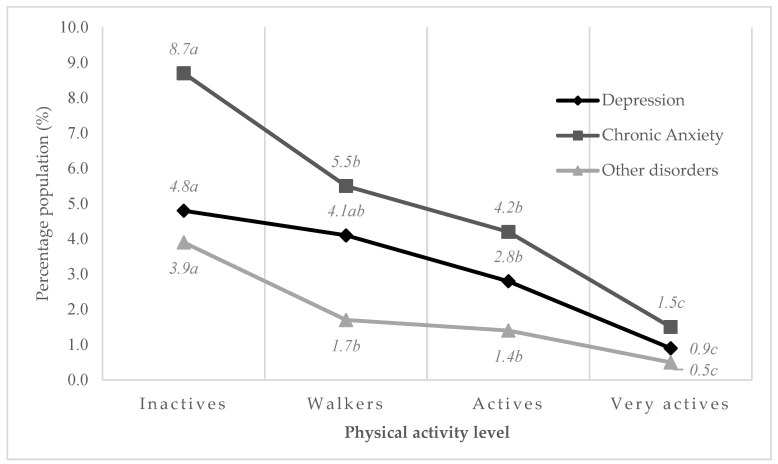
Relationship between prevalence of mental disorders and physical activity level (*p* < 0.001). a, b, c (Different subscript indicate significant differences in proportions. *p* < 0.05. Pairwise z-test).

Another of the main findings of this study was the dependency relationship found between the level of PA and the prevalence of depression, chronic anxiety, and other mental illnesses (*p* < 0.001). Active and Very active PA groups were found to be related to lower prevalence of the three mental pathological conditions analysed in this study (Figure 1).

Highest prevalence was found in the group of young people with Inactive PA level: depression (8.7%), chronic anxiety (4.8%), and other mental disorders (3.9%); physical inactivity seemed to be related to a higher predisposition to experience mental disorders in Spanish youth, something that has already been found in other studies, where physical inactivity was related to an increased risk of suffering anxiety [26,48].

The Walkers PA group presented lower prevalence of anxiety (8.7% vs. 5.5%. *p* < 0.05) and other mental disorders (3.9% vs. 1.7%. *p* < 0.05) than Inactives. Walkers and/or moderate/intense PA could help to reduce the prevalence of these mental pathologies, especially when performed in a natural environment or socialising with other people, as suggested by other studies consulted [49,50].

To find lower prevalence of depression, a higher level of PA might be needed, incorporating moderate and/or intense activities. The Very active PA group presented the lowest prevalence, with significant differences as compared to the rest of the level groups, so this condition could have a preventive action against the appearance of mental pathologies. Moderate and intense exercise has been found to be effective in reducing depression, with intense activity being more beneficial [27,51,52].

Along these lines, the calculated ORs and RRs in Inactives for mental disorders were compared to the groups with higher levels of PA. The main finding was an increased risk of mental disorders in the Inactives, especially compared to the Very active group.

The odds ratio for depression among Inactives was 5.35 compared to the Very active group, with a relative risk of 2.06. Even higher ORs were found in the 25–35 age group (OR: 5.46) compared to the 15–24 age group (OR: 4.39) although not the RRs.

Similar findings, with even higher ORs, were found for anxiety (OR: 6.14. RR: 2.15) and other mental disorders (OR: 8.52. RR: 2.25). A high or very high level of PA could reduce the risk or reduce the prevalence of mental pathologies [53] in Spanish young people. Similar to these findings, Brazilian young adults have been found to have increased ORs for depression (OR: 2.53), anxiety (OR: 2.18), and stress (OR: 1.75) in inactive compared to active young adults [54].

This study confirmed the association between PI and mental disorders in Spanish young people. Thus, PI was associated to higher prevalence of mental disorders in Spanish youth. Therefore, activities like walking or having a physically active lifestyle may help to prevent mental disorders or reduce the risk of suffering mental illnesses.

### 4.4. Limitations

This research had the limitations inherent to cross-sectional studies, presenting difficulties in interpreting the associations found. It would be advisable to go deeper into its findings through other research that allows causal relationships to be established. In new research, it would be advisable to include objective PA data of the participants, using inertial devices as well as conducting studies that allow us to find the minimum and optimal activity for the improvement of mental health in young people as well as including other methods for collecting participants’ experiences, such as online photovoice (OPV) [55].

### 4.5. Implications

Health promotion and education campaigns among young people could help to reduce the prevalence of mental disorders in the youth population. Increasing/promoting the level of PA in inactive young people or those with low levels of PA from inactivity or walking once a week to three 30-min sessions of moderate/intense activity would help to reduce the OR/RR of developing depression or anxiety in youth.

Increasing the hours of physical education in high schools and promoting active breaks in universities and workplaces could reduce the prevalence of mental disorders. In case of confinement, effective campaigns for increased physical activity would be necessary, aiming, among other objectives, to prevent depression and anxiety

## 5. Conclusions

Depression and chronic anxiety were more prevalent in young Spanish women, while the incidence of other mental disorders was higher in men. Associations were found between these pathologies and sex.

The level of PA was found to be related, through dependency relationships, to the prevalence of depression, anxiety, and other mental disorders in Spanish youth between 15 and 35 years of age.

Physical inactivity was found to be related to higher prevalence across PA level groups.

Prevalence of anxiety and other mental disorders was lower in the physical inactivity group than in the other level groups, even in those who only walked. However, the lowest prevalence of depression and chronic anxiety was found in the Very active group.

Walking did not seem to be sufficient to reduce the prevalence of depression in Spanish youth. In contrast, the Actives or Very actives PA levels did show reduced prevalence compared to the two groups below. The Very active PA-level group showed the lowest prevalence of depression.

Physical inactivity was associated with increased risk of mental disorders in Spanish youth compared to higher levels of PA. Relative to Very active PA level, inactivity had elevated ORs and RRs for depression, chronic anxiety, and other mental disorders, increasing even more in the older age group.

Increasing the level of PA among Spanish youth could help to reduce the prevalence of depression, chronic anxiety, and other mental disorders in this population.

## Figures and Tables

**Table 1:** Descriptive statistics of age, age group, and physical activity level and prevalence of depression, chronic anxiety, and other mental disorders of Spanish young population from ENSE 2017 outcomes.

Age (Years)	Total = 4195	Men = 2013	Women = 2182	*p*
Median (IQR)	27 (11)	27 (12)	27 (11)	0.114
Age Group (Years)	Total *n* (%)	Men *n* (%)	Women *n* (%)	p *
15–24	1640 (39.1)	835 (41.5) ^a^	805 (36.9) ^b^	<0.005
25–35	2555 (60.9)	1178 (58.5) ^a^	1377 (63.1) ^b^	
Depression	Total = 4195	Men = 2013	Women = 2182	p *
Yes *n* (%)	132 (3.1)	44 (2.2) ^a^	88 (4.0) ^b^	<0.005
No *n* (%)	4063 (96.9)	1969 (97.8) ^a^	2094 (96.0) ^b^	
Chronic anxiety	Total = 4195	Men = 2013	Women = 2182	p *
Yes *n* (%)	196 (4.7)	62 (3.1) ^a^	134 (6.1) ^b^	<0.001
No *n* (%)	3999 (95.3)	1951 (96.9) ^a^	2048 (93.9) ^b^	
Other mental disorders	Total = 4195	Men = 2013	Women = 2182	p *
Yes *n* (%)	68 (1.6)	41 (2.0) ^a^	27 (1.2) ^b^	<0.05
No *n* (%)	4127 (98.4)	1972 (98.0) ^a^	2155 (98.8) ^b^	
Physical activity level	Total = 4195	Men = 2013	Women = 2182	p *
Inactives	519 (12.4)	216 (10.7) ^a^	303 (13.9) ^b^	<0.001
Walkers	1511 (36.0)	529 (26.3) ^a^	982 (45.0) ^b^	
Actives	1311 (31.3)	691 (34.3) ^a^	620 (28.4) ^b^	
Very actives	854 (20.4)	577 (28.7) ^a^	277 (12.7) ^b^	

IQR, Interquartile Range; *n*, Number of participants; %, percentage; IAF, Physical Activity Index; Inactives, IAF = 0, report not walking at least 10 min in a row, one or more days a week, in item p.117; Walkers, IAF = 0, report walking at least more than 10 min at a time, one or more days a week; Actives, IAF between 1 and 30; Very actives, IAF greater than 30; ^a^^,^^b^, different superscript indicate significant differences between column proportions with significance level less than 0.05 in Levene’s z-test; *p*, *p*-value in Mann–Whitney U-test; *p* *, *p*-value chi-square test; Yes, report having experienced: depression (p.25.20A), chronic anxiety (p.25.21A), and other mental disorders (p.25.22A) in the ENSE 2017; No, negative responses to the above items.

**Table 2:** Relationships between the level of physical activity and the prevalence of depression, chronic anxiety, and other mental disorders in the Spanish youth population aged 15–35 years in the ENSE 2017.

Depression				
Physical activity level	Total= 4195	Yes = 132	No = 4063	*p*
Inactives	519 (12.4)	25 (4.8) ^a^	494 (95.2) ^a^	<0.001
Walkers	1511 (36.0)	62 (4.1) ^a,b^	1449 (95.9) ^a,b^	
Actives	1311 (31.3)	37 (2.8) ^b^	1274 (97.2) ^b^	
Very actives	854 (20.4)	8 (0.9) ^c^	846 (99.1) ^c^	
Chronic Anxiety				
Physical activity level	Total = 4195	Yes = 196	No = 3999	*p*
Inactives	519 (12.4)	45 (8.7) ^a^	474 (91.3) ^a^	<0.001
Walkers	1511 (36.0)	83 (5.5) ^b^	1428 (94.5) ^b^	
Actives	1311 (31.3)	55 (4.2) ^b^	1256 (95.8) ^b^	
Very actives	854 (20.4)	13 (1.5) ^c^	841 (98.5) ^c^	
Other Mental Disorders				
Physical activity level	Total= 4195	Yes = 68	No = 4127	*p*
Inactives	519 (12.4)	20 (3.9) ^a^	499 (96.1) ^a^	<0.001
Walkers	1511 (36.0)	25 (1.7) ^b^	1486 (98.3) ^b^	
Actives	1311 (31.3)	19 (1.4) ^b^	1292 (98.6) ^b^	
Very actives	854 (20.4)	4 (0.5) ^c^	850 (99.5) ^c^	

Data presented in absolute and relative frequencies; IAF, Physical Activity Index; Inactives, IAF = 0, report not walking, at least 10 min at a time one or more days a week, in item p.117; Walkers, IAF = 0, report walking, at least more than 10 min at a time, one or more days a week; Actives, IAF between 1 and 30; Very actives, IAF greater than 30; ^a–c^, different superscripts indicate significant differences between proportions in the same column with significance level less than 0.05 in Levene’s z-test; *p*, *p*-value chi-square test; Yes, report having experienced: depression (p.25.20A), chronic anxiety (p.25.21A), and other mental disorders (p.25.22A) in the ENSE 2017; No, negative answers to the previous items.

**Table 3:** OR and RR of depression, chronic anxiety, or other diseases in inactive young people compared to higher levels of physical activity surveyed in the ENSE 2017.

Or and RR: Depression							
	Physical Activity Level		OR	95% CI	RR	95% CI	*p*
Overall	Inactives	Walkers	1.18	0.74–1.90	1.13	0.81–1.59	0.489
		Actives	1.74	1.04–2.93	1.44	1.06–1.97	<0.05
		Very actives	5.35	2.39–11.96	2.06	1.67–2.52	<0.001
15–24 years	Inactives	Walkers	1.44	0.53–3.90	1.29	0.66–2.52	0.469
		Actives	2.88	0.92–9.05	1.94	1.09–3.47	0.058
		Very actives	4.39	1.09–17.75	2.13	1.32–3.43	<0.05
25–35 years	Inactives	Walkers	1.13	0.65–1.25	1.09	0.74–1.62	0.670
		Actives	1.47	0.82–2.65	1.29	0.90–1.87	0.193
		Very actives	5.46	2.02–14.78	1.93	1.55–2.41	<0.001
Or and RR: Chronic Anxiety							
			OR	95% CI	RR	95% CI	*p*
Overall	Inactives	Walkers	1.63	1.12–2.38	1.41	1.10–1.81	<0.05
		Actives	2.17	1.44–3.26	1.64	1.31–2.07	<0.001
		Very actives	6.14	3.28–11.50	2.15	1.84–2.52	<0.001
15–24 years	Inactives	Walkers	1.24	0.56–2.75	1.16	0.66–2.04	0.605
		Actives	2.92	1.14–7.46	1.96	1.21–3.16	<0.05
		Very actives	5.01	1.52–16.48	2.23	1.52–3.27	<0.005
25–35 years	Inactives	Walkers	1.80	1.17–2.76	1.50	1.14–1.98	<0.01
		Actives	1.95	1.24–3.08	1.53	1.18–1.99	<0.005
		Very actives	6.02	2.86–12.68	2.00	1.69–2.38	<0.001
Or and RR: Other Mental Disorders							
			OR	95% CI	RR	95% CI	*p*
Overall	Inactives	Walkers	2.38	1.31–4.33	1.77	1.26–2.47	<0.005
		Actives	2.73	1.44–5.15	1.84	1.34–2.52	<0.005
		Very actives	8.52	2.90–25.06	2.25	1.86–2.73	<0.001
15–24 years	Inactives	Walkers	2.40	0.98–5.90	1.77	1.07–2.92	<0.05
		Actives	3.29	1.25–8.65	2.08	1.30–3.31	<0.05
		Very actives	20.19	2.54–160.55	2.92	2.30–3.71	<0.001
25–35 years	Inactives	Walkers	2.36	1.06–5.24	1.76	1.12–2.77	<0.05
		Actives	2.41	1.03–5.61	1.70	1.11–2.61	<0.05
		Very actives	5.16	1.43–18.64	1.89	1.42–2.52	<0.01

OR, odds ratio; RR, relative risk; 95% CI, 95% confidence interval; *p*, *p*-value chi-square test; Overall, population aged 15–35 years; IAF, Physical Activity Index; Inactives, IAF = 0, report not walking at least 10 min at a time, one or more days a week, in item p.117; Walkers, IAF = 0, report walking at least more than 10 min at a time, one or more days a week; Actives, IAF between 1 and 30; Very actives, IAF higher than 30.

## Data Availability

The data used were obtained from public use files, which are available on the website of the Spanish Ministry of Health, Consumer Affairs, and Social Welfare: https://www.mscbs.gob.es/estadEstudios/estadisticas/encuestaNacional/encuesta2017.htm (accessed on 5 September 2021).
